# CMIP6 climate models imply high committed warming

**DOI:** 10.1007/s10584-020-02849-5

**Published:** 2020-09-04

**Authors:** Chris Huntingford, Mark S. Williamson, Femke J. M. M. Nijsse

**Affiliations:** 1 Centre for Ecology and Hydrology, Wallingford, Oxfordshire, OX10 8BB UK; 2 Global Systems Institute, University of Exeter, Exeter, EX4 4QE UK; 3 College of Engineering, Mathematics and Physical Science, University of Exeter, Exeter, UK

Current climate change is highly transient, and Earth is at a significant distance from thermal equilibrium. This well-established finding is predominantly due to the large flux of thermal energy currently entering the oceans. If globally the sources and sinks of atmospheric GHGs were to become zero, this is identical to stating that their concentrations are subsequently invariant. Such a policy would correspond to a full offset of the remaining emissions by both land and ocean CO_2_ drawdown, as well as any implementation of carbon capture and storage (CCS) methods. The 5th Intergovernmental Panel on Climate Change (IPCC) report defines this as a “constant composition commitment” (Collins et al., 2013).

With fixed atmospheric GHGs, the planet will move to an equilibrium state, at which time land and ocean CO_2_ offsets will tend to zero. Hence, without any deliberate CCS implementation, gross GHG emissions will then need to be negligible. However, an equilibrium state also implies thermal stability too, with global warming invariant. It is the difference between the observed transient planetary response and equilibrium climate state for the same GHG levels that characterise this extra warming. We analyse this additional warming for historical and current GHG concentrations, utilising ECS values for the new ESMs in the Coupled Model Intercomparison Project v6 (CMIP6).

The current lag in global warming behind an equilibrium state has been explored with the CMIP5 ensemble of ESM simulations, following the forcing protocol of Taylor et al. (2012). These calculations raise the prospect that even current atmospheric GHG levels commit to near-surface global warming likely greater than 1.5 °C above pre-industrial levels (and even higher over land; Huntingford and Mercado, 2016). With rising international discussion of minimising global emissions, which in some circumstances could correspond to invariant atmospheric GHG levels, there is renewed interest in the magnitude of stabilised global warming levels. Here, we perform a simplistic but illustrative analysis to define this lag, utilising the ECS values of the recent CMIP6 ESMs derived by established methods (Gregory et al., 2004).

The ECS value for each climate model is the ESM-specific projected warming for a doubling of atmospheric CO_2_. Other radiatively active atmospheric gases, including non-CO_2_ greenhouse gases, are compared by their impact on radiative forcing, leading to a single aggregated statistic of carbon dioxide equivalent concentrations, CO_2_e (ppm). The radiative response to increasing CO_2_ is logarithmic, and the CO_2_e calculation accounts for this. Knowledge of ECS allows a scaling to estimate committed equilibrium warming, Δ*T*_Com_ (°C), for any given CO_2_e level, by the simple statistic of ${\Delta  T}_{\mathrm{C}\mathrm{om}}=\log \left[\frac{{\mathrm{C}\mathrm{O}}_2\mathrm{e}}{\mathrm{C}{\mathrm{O}}_2{\mathrm{e}}_{\mathrm{PI}}}\ \right]\times \frac{\mathrm{ECS}}{\log 2}$ where “PI” is pre-industrial. Hence committed warming describes the eventual warming in the event of no further gas concentration changes.

Here, we derive Δ*T*_Com_ for different ECS values (Fig. 1), using available estimates of historical CO_2_e values (Meinshausen et al., 2011), with CO_2_e_,PI_ as the CO_2_e value for the year 1860. The CO_2_e dataset we use is based on atmospheric measurements until the year 2005, followed by the RCP8.5 scenario (Meinshausen et al., 2011). The RCP8.5 scenario is a good approximation to actual GHG concentration changes over the last 15 years. The CO_2_e values used do not contain the effects of volcanoes or solar fluctuations.

**Fig. 1:**
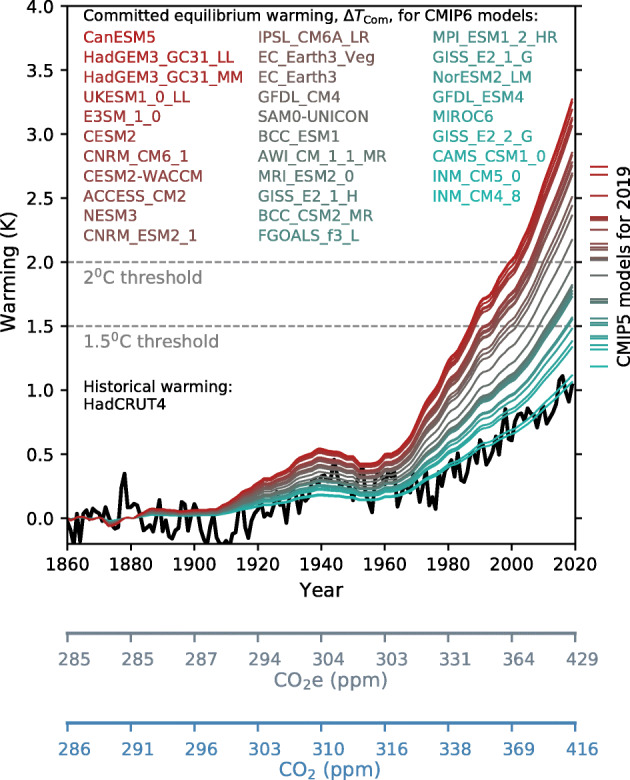
Committed equilibrium warming, Δ*T*_Com_, for the atmospheric concentrations of radiatively-active gases associated with each year since the pre-industrial period. Calculations combine ECS values of the CMIP6 ESMs with past CO_2_e values. ESMs are colour coded from hottest to coldest (red to green). The black curve is the measurement-based annual mean global temperature from the HadCRUT4 dataset, normalised to have a mean of zero between 1860 and 1899. Marked are the 1.5 °C and 2.0 °C warming thresholds. For comparison, the same calculations with the CMIP5 model ensemble, but for the year 2019 only, are marked on the right-hand side of the diagram. An additional horizontal axis shows the yearly CO_2_e values used to calculate Δ*T*_Com_. The non-monotonic increase, in time, of CO_2_e and Δ*T*_Com_ around year 1950 is due to the high atmospheric concentrations of cooling aerosols (see Meinshausen et al., 2011 for a description of historical atmospheric forcings). Also presented as an extra axis are the historical CO_2_ concentrations contributing to the CO_2_e values

Figure 1 shows that for many ESMs, there is a substantial difference between transient global warming levels (black curve) and their related committed equilibrium warming for the same CO_2_e levels (coloured curves). For 2019, even if atmospheric GHGs (and aerosols) were to remain at the levels for that year, there would be continued warming towards an equilibrium state that has a high chance of being above 1.5 °C, possibly exceeding 2.0 °C. Specifically, if regarding all models as equally likely at estimating global warming changes, then for CMIP6 and year 2019 radiative forcing, the chances of crossing the 2.0 °C threshold are 52% and crossing the 1.5 °C threshold are 84%. For CMIP5, the numbers are 50% and 81%, respectively. The endpoints of each curve (CMIP6) or right-hand marks (CMIP5) in Fig. 1 correspond to the values in these calculations. This extra warming suggests that to constrain warming at or below such thresholds may eventually require the massive implementation of technologies that can extract CO_2_ from the atmosphere. The time taken from the start of GHG invariance to being very near to equilibrium temperature is, though, of the order centuries-to-millennia (Meehl et al., 2007 p823; Li et al., 2013). However, if Earth is represented better by ESMs with a high ECS value, then, in particular, the 1.5 °C threshold will be met much sooner. We investigate this by operating a global two-box thermal model (Williamson et al., 2019; Geoffroy et al., 2013; Gregory, 2000) calibrated against the CMIP6 ensemble. These simulations are with radiative forcing prescribed as historical then steady from 2019 onwards, and the ECS values are identical to those leading to Fig. 1. For the models that warm sufficiently to cross the 1.5 °C threshold (and by year 2500; 23 models), then the median year of reaching that warming level is soon, at 2025. For models that cross the 2.0 °C threshold (again by year 2500; 16 models), then the median year of attaining that level is 2101.

There are caveats associated with our analysis. ESMs are not weighted by any performance metrics, and in instances where two or more models are from the same research centre, this may cause similarities in projection. There is an assumption that in the CO_2_e calculation, the radiative forcings associated with different radiatively active gases are well-known. In particular, there are known uncertainties in the strength of the negative cooling strength of aerosols (Yu et al., 2006), noting that it is their variation that creates the non-monotonic behaviour of CO_2_e between 1940 and 1960 (Fig. 1). If clean air acts massively reduce atmospheric aerosol concentrations, but greenhouse gases are invariant, then Δ*T*_Com_ values maybe even higher. The analysis takes no account of some recent advances that link features of current interannual temperature fluctuations, via the emergent constraint technique, to ECS. For instance, some (Cox et al., 2018) use this approach to argue that ECS values are likely towards the lower part of the range suggested by the CMIP5 model ensemble. Furthermore, the scaling used to calculate Δ*T*_Com_ has an implicit assumption that ECS is an invariant quantity, which some have recently challenged (Gregory et al., 2020). Rugenstein et al. (2019a) find that millennial-length simulations indicate that ECS may be of order 17% higher than that inferred from the analysis of initial centuries of such ESM calculations and so implies that any final global temperature under fixed gas concentrations may be even higher than those of Fig. 1. In more general terms, Rugenstein et al. (2019b) explore multiple millennial-timescale simulations that do exist and as performed with ESMs. These projections are often initialised with an abrupt conceptual jump in atmospheric CO_2_, and an opportunity ahead might be for research centres to contribute to a standardised ensemble of historical calculations followed by long commitment simulations for fixed and contemporary GHG concentrations. More availability of ESM simulations with fixed climate forcings that follow on from realistic modelling of the historical period will provide highly valuable information on the transient features of the Earth system. The raised understanding generated by such calculations will also support regional assessment of change and so beyond that of just global temperature variation. For instance, Sigmond et al. (2020) show (for stabilised forcings applied to two ESMs) that the Atlantic Meridional Overturning Circulation (AMOC) will continue to adjust for many centuries, even though its end state is nearly independent of final fixed warming level.

We have asked the illustrative but specific question of should atmospheric greenhouse gases suddenly stop increasing, what additional global warming will occur based on current understanding? Such a constant composition commitment is less ambitious than the recent aspiration of many to achieve “net-zero” global emissions of GHGs. Net-zero has been generally defined as not including natural sinks and is only achieved when anthropogenic CO_2_ emissions are balanced globally by anthropogenic removals. For instance, the IPCC report on constraining global warming to 1.5 °C (IPCC, 2018) states that: “Reaching and sustaining net zero global anthropogenic CO_2_ emissions and declining net non-CO_2_ radiative forcing would halt anthropogenic global warming on multi-decadal time scales (high confidence)”. Implementation of a net-zero policy may cause global temperatures to initially either increase or decrease (Allen et al., 2018; p64) depending on the balance between the transient warming effects we discuss and any offsetting cooling predominantly by natural CO_2_ drawdown. Schleussner et al. (2016; p832) note that to stabilise at low temperature thresholds, and in particular 1.5 °C, is likely to require global temperature decreases. Schleussner et al. (2016) argue that transient effects, following a period of global warming overshoot, require a much better understanding. The “net-zero” ambition was derived from Article 4 of the Paris climate agreement (COP21, 2015), although there remain many open questions surrounding its precise interpretation (Fuglestvedt et al., 2018). The focus of this study, however, has been the lower ambition of achieving fixed GHG concentrations that, by definition, allows for a continuation of some positive net emissions, which are balanced by natural sinks.

In summary, climate researchers are very aware of the difference between transient warming and equilibrium temperature levels. However, in general, there is often a misunderstanding in society, corresponding to a belief that achieving constant atmospheric GHG composition implies that global mean temperatures will not change from that point forward. Figure 1 characterises transient versus equilibrium warming differences utilising the latest generation of climate models.

## Data Availability

The CO_2_e time series is from the RCP scenario site, selecting the rcp85 file: http://www.pik-potsdam.de/~mmalte/rcps/.

## References

[CR1] Allen, M.R., Dube, O.P., Solecki, W., Aragón-Durand, F., Cramer, W., Humphreys, S., Kainuma, M., Kala, J., Mahowald, N., Mulugetta, Y., Perez, R., Wairiu, M. and Zickfeld, K. (2018) Framing and Context. In: IPCC (2018) Masson-Delmotte, V. *et al* (Eds) Global warming of 1.5°C. An IPCC special report on the impacts of global warming of 1.5°C above pre-industrial levels and related global greenhouse gas emission pathways, in the context of strengthening the global response to the threat of climate change, sustainable development, and efforts to eradicate poverty. World Meteorological Organization, Geneva, Switzerland, pp 49–91

[CR2] Collins M, Knutti R, Arblaster J, Dufresne J-L, fichefet T, Friedlingstein P, Gao X, Gutowski WJ, Johns T, Krinner G, Shongwe M, Tebaldi C, Weaver AJ, Wehner M (2013) In: Stocker TF et al (eds) Climate change 2013: the physical science basis. Contribution of Working Group I to the Fifth Assessment Report of the Intergovernmental Panel on Climate Change. Cambridge University Press, Cambridge UK and New York USA, pp 1029–1136

[CR3] COP21 (2015) Adoption of The Paris Agreement. Proposal by the President. FCCC/CP/2015/L.9/Rev.1, Paris Climate Change Conference – November 2015, COP 21, United Nations Framework Convention on Climate Change, Paris, available at: https://unfccc.int/resource/docs/2015/cop21/eng/l09r01.pdf

[CR4] Cox PM, Huntingford C, Williamson MS (2018) Emergent constraint on equilibrium climate sensitivity from global temperature variability. Nature 553:319−+. 10.1038/nature25450 10.1038/nature25450 29345639

[CR5] Fuglestvedt J, Rogelj J, Millar RJ, Allen M, Boucher O, Cain M, Forster PM, Kriegler E, Shindell D (2018). Implications of possible interpretations of ‘greenhouse gas balance’ in the Paris agreement. Phil Trans R Soc A.

[CR6] Geoffroy O, Saint-Martin D, Olivié DJL, Voldoire A, Bellon G, Tytéca S (2013). Transient climate response in a two-layer energy-balance model. Part 1: analytical solution and parameter calibration using CMIP5 AOGCM experiments. J Clim.

[CR7] Gregory JM (2000). Vertical heat transports in the ocean and their effect an time-dependent climate change. Clim Dyn.

[CR8] Gregory JM, Ingram WJ, Palmer MA, Jones GS, Stott PA, Thorpe RB, Lowe JA, Johns TC, Williams KD (2004). A new method for diagnosing radiative forcing and climate sensitivity. Geophys Res Lett.

[CR9] Gregory JM, Andrews T, Ceppi P, Mauritsen T, Webb MJ (2020). How accurately can the climate sensitivity to CO_2_ be estimated from historical climate change?. Clim Dyn.

[CR10] Huntingford C, Mercado LM (2016). High chance that current atmospheric greenhouse concentrations commit to warmings greater than 1.5 °C over land. Scientific reports.

[CR11] IPCC (2018) Masson-Delmotte, V. *et al.* (Eds). Global warming of 1.5°C. an IPCC special report on the impacts of global warming of 1.5°C above pre-industrial levels and related global greenhouse gas emission pathways, in the context of strengthening the global response to the threat of climate change, sustainable development, and efforts to eradicate poverty. World Meteorological Organization, Geneva, Switzerland

[CR12] Li C, von Storch J-S, Marotzke J (2013). Deep-ocean heat uptake and equilibrium climate response. Clim Dyn.

[CR13] Meehl GA, Stocker TF, Collins WD, Friedlingstein P, Gaye AT, Gregory JM, Kitoh A, Knutti R, Murphy JM, Noda A, Raper SCB, Watterson IG, Weaver AJ, Zhao Z-C, Solomon S (2007). Climate change 2007: the physical science basis.

[CR14] Meinshausen M (2011). The RCP greenhouse gas concentrations and their extensions from 1765 to 2300. Clim Chang.

[CR15] Rugenstein M, Bloch-Johnson J, Gregory J, Andrews T, Mauritsen T, Li C, Frölicher TL, Paynter D, Danabasoglu G, Yang S, Dufresne J-L, Cao L, Schmidt GA, Abe-Ouchi A, Geoffroy O, Knutti R (2019). Equilibrium climate sensitivity estimated by equilibrating climate models. Geophysical Research Letters.

[CR16] Rugenstein M, Bloch-Johnson J, Abe-Ouchi A, Andrews T, Beyerle U, Cao L, Chadha T, Danabasoglu G, Dufresne J-L, Duan L, Foujols M-A, Frölicher T, Geoffroy O, Gregory J, Knutti R, Li C, Marzocchi A, Mauritsen T, Menary M, Moyer E, Nazarenko L, Paynter D, Saint-Martin D, Schmidt GA, Yamamoto A, Yang S (2019). LongRunMIP - motivation and design for a large collection of millennial-length GCM simulations. Bull Am Meteorol Soc.

[CR17] Schleussner C-F, Rogelj J, Schaeffer M, Lissner T, Licker R, Fischer EM, Knutti R, Levermann A, Frieler K, Hare W (2016). Science and policy characteristics of the Paris agreement temperature goal. Nat Clim Chang.

[CR18] Sigmond M, Fyfe JC, Saenko OA, Swart NC (2020). Ongoing AMOC and related sea-level and temperature changes after achieving the Paris targets. Nat Clim Chang.

[CR19] Taylor KE, Stouffer RJ, Meehl GA (2012). An overview of the cmip5 and the experiment design. Bull Am Meteorol Soc.

[CR20] Williamson MS, Cox PM, Nijsse FJMM (2019). Theoretical foundations of emergent constraints: relationships between climate sensitivity and global temperature variability in conceptual models. Dynamics and Statistics of the Climate System.

[CR21] Yu H, Kaufman YJ, Chin M, Feingold G, Remer LA, Anderson TL, Balkanski Y, Bellouin N, Boucher O, Christopher S, DeCola P, Kahn R, Koch D, Loeb N, Reddy MS, Schulz M, Takemura T, Zhou M (2006). A review of measurement-based assessments of the aerosol direct radiative effect and forcing. Atmos Chem Phys.

